# Functions of Matricellular Proteins in Dental Tissues and Their Emerging Roles in Orofacial Tissue Development, Maintenance, and Disease

**DOI:** 10.3390/ijms22126626

**Published:** 2021-06-21

**Authors:** Georgia Nikoloudaki

**Affiliations:** 1 Schulich Dentistry Department, Schulich School of Medicine and Dentistry, Western University, London, ON N6A 5C1, Canada; gnikolou@uwo.ca; Tel.: +1-519-661-2111 (ext. 81102); 2 Department of Anatomy and Cell Biology, Schulich School of Medicine and Dentistry, Western University, London, ON N6A 5C1, Canada

**Keywords:** matricellular proteins, periostin, SPARC, SIBLINGs, DSPP, DMP1, tenascin, dental tissues, tooth development, oral carcinoma

## Abstract

Matricellular proteins (MCPs) are defined as extracellular matrix (ECM) associated proteins that are important regulators and integrators of microenvironmental signals, contributing to the dynamic nature of ECM signalling. There is a growing understanding of the role of matricellular proteins in cellular processes governing tissue development as well as in disease pathogenesis. In this review, the expression and functions of different MP family members (periostin, CCNs, TSPs, SIBLINGs and others) are presented, specifically in relation to craniofacial development and the maintenance of orofacial tissues, including bone, gingiva, oral mucosa, palate and the dental pulp. As will be discussed, each MP family member has been shown to have non-redundant roles in development, tissue homeostasis, wound healing, pathology and tumorigenesis of orofacial and dental tissues.

## 1. Introduction

In recent years, a plethora of research has demonstrated that the extracellular matrix (ECM) composition is an important determinant of tissue development and organization, tissue homeostasis and remodelling, and progression of disease [1]. While ECM structural components fibrin, collagen and fibronectin provide mechanical support, matricellular proteins (MCPs) are important regulators and integrators of microenvironmental signals, contributing to the dynamic nature of ECM signalling [2]. As a class of proteins, MCPs specifically modulate cell-matrix interactions and cell function (adhesion, spreading, migration, proliferation and differentiation) [3] by interacting with cell surface receptors (e.g., integrins), other bioeffector molecules, and structural matrix proteins.

MCPs act in a tightly controlled temporal and context-specific manner to guide different aspects of tissue development, response to injury and tumorigenesis [4], but their expression is generally low in adult tissues under physiologic conditions [5,6,7,8]. As most MCPs are not involved in tissue homeostasis, it is understandable that analysis of these proteins through genetic deletion revealed mice developed normally and exhibited no major phenotypic changes in adulthood [9,10]. However, loss of MCPs is associated with a wide range of alterations in injured and remodelling tissues where specific MCPs and their roles in healing are context-dependent [5,11,12,13]. On the other hand, CCN2-knockout mice die shortly after birth, while CCN1 and CCN5 constitutive knockout mice are embryonic lethal, showing that these proteins are essential for development [14,15].

This review outlines what is presently known about the role of the MCP families Gla-containing proteins (periostin, βigh3, matrix Gla protein), CCN, thrombospondin, SPARC, SIBLINGs, and tenascin in orofacial and dental tissue development, wound healing and neoplastic disease, and mechanisms involved in their regulation.

## 2. Gla-Containing ECM Proteins

The Gla protein family members contain vitamin K-dependent g-carboxyglutamic acid residues, which have a high affinity for calcium ions, thus conferring important roles in coagulation and bone homeostasis [16]. The most studied members of this family in orofacial tissues are periostin (Pstn), βιgh3 and matrix Gla protein.

### 2.1. Periostin

Periostin (Pstn) was initially identified as an 811 amino acid protein secreted by murine osteoblasts that were required for cell adhesion [17]. Originally, it was termed osteoblast-specific factor 2 (OSF-2) due to its localization in proximity to the periodontal ligament and periosteum of mice; it was renamed periostin in 1999 and subsequently classified as a matricellular protein in 2007 by Norris and Colleagues [18]. Structurally, periostin is a secreted 90 kDa N terminus-glycosylated protein with a multi-domain structure, including amino-terminal EMI domain, four tandem fascilin domains, and a carboxyl-terminal domain [19], with several different isoforms described [20,21,22,23]. This complex domain structure allows for numerous interactions with extracellular/secretory proteins [24], via which exerts its multiple functions. The physiological significance of periostin expression in adult systems has been mainly identified in collagen-rich biomechanically active tissues [8], highlighting a subtle but complex role for periostin in tissue homeostasis, healing and pathology. In terms of its physiological function, periostin modulates crosslinking and stabilization of the extracellular matrix, including collagen fibrillogenesis [18,25] and providing a scaffold for the assembly of several extracellular matrix proteins (type I collagen, fibronectin, tenascin-C, and laminin γ2) and accessory proteins (BMP-1 and CCN3) [26,27], although the binding site for collagens has not been yet identified [18,24].

#### 2.1.1. Development

In the oral cavity, periostin is expressed both in embryonic, developing and postnatal orofacial and dental tissues.

In developing teeth, mRNA and protein PSTN are asymmetrically localized to the lingual/palatal and buccal regions during early epithelial-mesenchymal interaction sites, where it could be linked with deposition and organization of other ECM adhesion molecules [28,29]. During tooth morphogenesis, at the cap and early bell stage of the tooth germs, periostin is expressed at the interface between the inner enamel epithelium and pre-odontoblasts, as well as in mesenchymal tissues around the cervical loop and dental follicles, which then disappear in advance of tooth development. During the late bell and mineralization phases of tooth development, periostin is expressed in dental papilla cells and trans-differentiating odontoblasts [30].

#### 2.1.2. Postnatal

**Figure 1:**
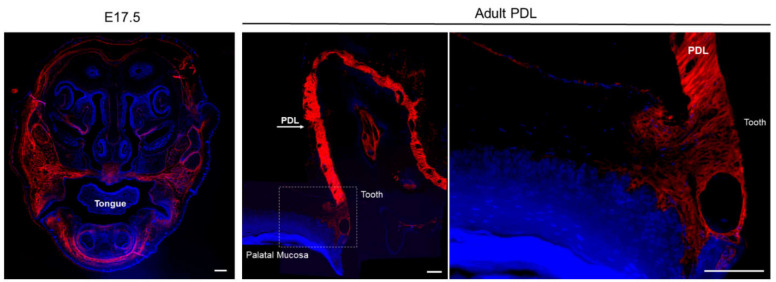
Immunofluorescent staining for periostin (red) in murine embryonic and adult orofacial tissues. The area highlighted in the rectangle is magnified on the right. An intense periostin signal is detected on the developing secondary palate at E17.5 and at the periodontal ligament (PDL) of adult mice. (Anti-Periostin sc49480, 1:100, Santa Cruz Biotechnology) Scale bar: 200 μm.

In the adult rodent, periostin expression is predominantly identified in the periodontium (Figure 1). Immunoelectron microscopic observation of the mature periodontal ligament (PDL) verified the localization of periostin between the cytoplasmic processes of periodontal fibroblasts and cementoblasts and the adjacent collagen fibrils. This finding suggests that periostin participates in various cellular events that are involved in maintaining the integrity of adult teeth, particularly at the sites of hard–soft tissue interface, serving as adhesive equipment for bearing mechanical forces, including occlusal force and tooth eruption. [29,30]. In addition, in situ hybridization and immunohistochemical analysis of rodent mandibles revealed the presence of periostin on the alveolar bone surface [30]. The importance of periostin in the maintenance of PDL integrity is also highlighted by studies using transgenic mice where periostin is genetically deleted. Although periostin knockout (Pstn^−/−^) mice are viable, the deletion results in defective collagen fibrillogenesis due to reduction in fibril diameter and defective collagen crosslinking [18,31], resulting in loss of architecture and functional disruption of several collagenous-based tissues, particularly those subject to constant mechanical loading, such as the PDL, heart, and bone [32]. Micro-computed topography imaging analysis on Pstn^−/−^ mice skulls revealed the early onset of severe periodontal disease, a significant reduction in bone density, structural defects in the incisors resulting in enhanced tooth wear, and showed that orbital bones are completely missing or fail to fuse properly [8]. Removal of masticatory forces reduced this damage, showing that the loss of periostin likely impairs the ability of the tissue to withstand mechanical loading [9]. Additionally, the PDL of Pstn^−/−^ mice demonstrated an uneven, irregular distribution of several crucial ECM proteins, such as type I collagen, fibronectin, and tenascin-C [33].

Periostin also plays a role in controlling postnatal tooth formation. In wild-type mice, periostin is expressed in pre-odontoblasts, odontoblasts and ameloblasts, but genetic deletion of periostin in Pstn^−/−^ mice resulted in a massive increase in dentin formation after mastication and significant enamel defects [32]. Deletion of periostin also resulted in the alteration of expression profiles of many non-collagenous proteins such as dentin sialophosphoprotein (DSPP), dentin matrix protein 1 (DMP1), bone sialoprotein (BSP), and osteopontin (OPN) in incisor dentin [34], as well as to morphological changes of the osteocytes (from spindle to round shape) in the jaws as shown by acid-etched scanning electron microscopy [35]. FAM20C, a member of a “family with sequence similarity 20”, also known as DMP4 in mice [36], is required for the maintenance of healthy periodontal tissues and functions as an intracellular protein kinase in the secretory pathway [37]. Recently it was shown that FAM20C directly bounds to periostin via the Fas-I domain and phosphorylates periostin in vitro. In addition, immunohistochemical analysis demonstrated that immunolocalization of FAM20C in murine PDL overlapped with that of periostin [38]. Taken together, periostin plays a crucial role in the remodelling of collagen matrix and induction of various non-collagenous proteins in dental tissues, showing that periostin is critical for the postnatal tooth and periodontium development, as well as the maintenance of PDL integrity [29,39].

#### 2.1.3. Orthodontic Movement

In adult rodents, during physiological tooth movement, periostin is localized in the PDL surrounding the mesial and distal roots of molars [40]. Mechanical loading maintains sufficient periostin expression to ensure the integrity of the periodontium in response to occlusal load [9]. Pstn^−/−^ mice exhibited significant damages to the periodontal tissues following exposure to a masticatory force, but placing the animals on a soft diet was sufficient to reduce the mechanical strain on the PDL and could partially rescue both the enamel and periodontal disease-like phenotypes [9]. On the contrary, in the absence of mechanical stress, the PDL of Wistar rats undergoes degradation concomitantly with a marked decrease in periostin and connective tissue growth factor (CTGF) mRNA levels in the PDL [41], suggesting that periostin is essential for maintaining the integrity of the PDL under physiological occlusal and mastication forces.

Orthodontic tooth movement is a process in which the application of a mechanical force induces bone resorption on the pressure side and bone apposition on the tension side [42]. During orthodontic movement, periostin is involved in the process of periodontium remodelling in response to mechanical force [41,43,44]. Application of orthodontic forces induced divergent changes of periostin mRNA levels between the pressure and tension sites of rat PDL, with increased levels in the compressed side of the ligament when compared to tension sites [40]. In Pstn^−/−^ mice, during application of orthodontic forces, the distance of the tooth movement and mineral deposition rates were significantly reduced when compared to WT mice, exhibiting impaired arrangement, digestion, and integrity of collagen fibrils. The authors also reported a marked reduction in the number of osteoclasts, as reflected by the number of tartrate-resistant acid phosphatase-positive cells and reduced expression of both RANKL (a potent stimulator of osteoclasts) and osteoprotegerin (a strong inhibitor of osteoclasts) in Pstn^−/−^ mice when compared to WT control, which could contribute in part for the delayed bone remodeling during tooth movement [45]. Further, immunolocalization of collagenolytic enzymes such as cathepsin K and matrix metalloproteinase-1 and -2 (MMP1, MMP2) was significantly decreased after orthodontic force application in the compressed side of PDL of Pstn^−/−^ mice compared with WT mice [43]. Taken together, it is apparent that periostin is essential for the periodontium in response to orthodontic tooth movement; however, further studies are required to elucidate its role and functions during this process.

#### 2.1.4. Wound Healing and Inflammation

Extracellular matrix molecules are actively remodeled during wound healing and chronic inflammatory diseases, such as periodontitis [44]. Wound healing in soft connective tissues, such as the oral mucosa and the gingival tissue, is defined as a coordinated series of overlapping phases: hemostasis, inflammation, proliferation and remodeling, concomitant with epithelial barrier formation, which lead to the resolution of the injury [46]. Myofibroblasts are key cells during wound healing, responsible for extracellular matrix (ECM) synthesis, remodeling and tissue contraction [47,48]. Transforming growth factor-β (TGF-β), which is produced in the early stages of wound healing, is known to cause an increase in α-SMA expression through phosphorylation of Smad3 and plays a major role in myofibroblast differentiation [49]. Recently, Marconi and colleagues also showed that when human gingival fibroblasts (hGFs) are treated with TGFβ in vitro, they differentiate into myofibroblasts, determined by morphological and molecular modifications such as the induction of α-SMA, Vimentin, E-cadherin, β-catenin, and Smad 2/3 [50]. After the formation of actin-myosin contractile bundles, stress fibres, it is the neo-expression and incorporation of α-SMA that significantly augments the contractile activity of activated myofibroblasts [51,52].

The oral mucosa and the gingival tissues typically heal with minimal scarring, with many similarities evident to the healing of fetal tissue [53,54]. While periostin has been shown to regulate myofibroblast differentiation, matrix synthesis and re-epithelialization in the skin [7,19,55,56], during gingival healing, a very low level of myofibroblasts is observed, suggesting that adoption of a contractile myofibroblast phenotype is not a significant event in gingival wound healing [57], providing a potential explanation of the scarless healing of the latter. More specifically, using a gingivectomy model in rats, it has been shown that periostin regulates ECM synthesis, upregulating fibronectin and collagen synthesis via integrin β1, FAK and JNK, but it is not associated with myofibroblast differentiation during gingival wound healing [57]. The low number of myofibroblasts, as shown by the absence of α-smooth muscle immunoreactivity in the wounded tissues, is postulated as an underlying reason for the reduced scar formation in the healing of the gingival tissue [57]. In vitro, treatment with exogenous recombinant human periostin (rhPN) to gingival fibroblasts derived from human tissues resulted in increased fibronectin and collagen synthesis, an effect that was attenuated by pharmacological inhibition of FAK and JNK signalling. In support of the in vivo observations, the addition of rhPN did not induce myofibroblast differentiation or proliferation of gingival fibroblasts, in contrast to its effect on skin and hypertrophic scar fibroblasts as reported by other studies [55,56,58]. In addition, gingival fibroblasts predominantly attach to periostin through β1-integrins [57]. The inability of gingival fibroblasts to transition to myofibroblasts even in the presence of periostin may provide a possible explanation of scarless healing that is evident in attached gingiva compared to other tissues such as skin [59].

While gingival tissue typically heals without scarring, it is still associated with several fibrotic conditions, including drug-induced gingival enlargement (DIGE). DIGE is a pathological condition that develops as a side effect from the systemic administration of the antihypertensive drug nifedipine and anti-seizure drugs, and it is classified as a fibrotic lesion [60,61]. As such, it is characterized by an imbalance in the remodelling and deposition of ECM. Gingival connective tissue from patients with DIGE showed increased immunoreactivity of periostin when compared to healthy individuals, but the absence of α-smooth muscle actin (α-SMA) expressing cells [62,63]. In vitro, periostin mRNA and protein levels were upregulated in response to nifedipine treatment via a TGFβ−dependent mechanism [62]. Treatment of human gingival fibroblasts in vitro with different concentrations of phenytoin also resulted in increased periostin protein levels, which correlated with phosphor-Smad3 (p-SMAD3) phosphorylation, suggesting common mechanisms are responsible for DIGE irrespective of the drug [64]. Based on the fact that DIGE is defined as a fibrotic lesion, the molecular pathology is in direct contrast to skin scarring in that no differentiation or persistence of myofibroblast is associated with the condition.

The palatal mucoperiosteum, while still part of the oral environment, is associated with significant scarring in response to injury [65]. Using a full-thickness excisional palatal wound model, it was shown that periostin mRNA and protein levels are increased in response to injury, where it is associated with fibronectin production, myofibroblast differentiation and infiltration of macrophages to the wound site. In vitro, palatal fibroblasts isolated from Pstn^−/−^ mice exhibited reduced contractability, which was rescued by the exogenous addition of periostin, and reduced fibronectin synthesis. These effects were also modulated by the stiffness of the microenvironment via the integrin-β1/RhoA pathway, suggesting that periostin and the ECM stiffness act as important modulators of cell behaviour during palatal healing [66]. The contrasting effects of periostin among dermal, gingival and palatal tissues demonstrate the tissue-specificity of its bioactivity in relatively homologous tissues [59].

Controlled degradation of the ECM is essential in physiological situations involving connective tissue remodellings, such as tissue morphogenesis, repair, and angiogenesis. On the other hand, excessive breakdown of connective tissue components plays an important role in the destruction of functional tissue architecture, e.g., the irreversible destruction of periodontal support tissues in periodontitis, such as the alveolar bone, PDL, and root cementum leading to tooth loss [67]. The first to investigate the influence of spatial and temporal periodontal inflammation on the levels of the periostin in an inflammatory-induced periodontal disease model in vivo was Padial-Molina and colleagues in a series of animal and clinical studies [68]. Using a ligature periodontal inflammatory disease model in rats, they found that periostin immunoreactivity is reduced in the presence of inflammation on the PDL and is correlated with detrimental changes to the periodontium over time [68]. In vitro, hPDL cells had lower periostin protein levels when treated with TNFα and/or bacterial endotoxin. However, these changes were only observed when cells were cultured in a mechanically challenged environment but not in the un-loaded control condition, and the high variability among the groups with an unclear pattern makes it [69] difficult to draw conclusions [70]. When exogenous periostin was added to the experimental conditions, hPDL cells exhibited increased proliferation and migration [71]. Another study demonstrated that while IL-4 and IL-13 cytokines significantly induced periostin production on human periodontal ligament (hPDL) and human gingival fibroblasts (hGF) cultures, no stimulatory effect was observed by TNF-α or bacterial endotoxin on the production of periostin. Additionally, exogenous periostin did not have a significant effect on the production of inflammatory cytokines by hPDL or hGF cells, suggesting that although these cells may be a source of periostin in periodontitis lesions, its role in the inflammatory response or in matrix protein metabolism might be limited [72]. Tang et al., using human periodontal ligament stem cells (PDLSCs), investigated the role of periostin under inflammatory conditions in the periodontium and found that periostin promoted both the migration and osteogenic differentiation potential of PDLSCs via the JNK signalling pathway with TNFα treatment [69]. PDLSCs, among other stem cell populations isolated from the oral cavity, have attracted scientific attention for their use in bioengineering and clinical applications toward the repair and regeneration of wounded tissues. PDLSCs are not only easily obtainable, but also they have demonstrated excellent stability, plasticity, and differentiation ability to various types of cells, such as osteoblasts [73,74].

In clinical studies investigating periostin levels in the gingival crevicular fluid (GCF) [75,76] and Pstn mRNA in tissues [77] at different stages of periodontal disease, it was shown that the total amount and concentration of periostin in the GCF, as well as Postn mRNA levels, are reduced with the progression and severity of the disease from healthy controls to chronic periodontitis groups and correlate negatively with clinical parameters of disease [77]. When looking at the expression profile of periostin in GCF/wound fluid over time after periodontal surgery, it was shown that periostin levels increase after surgery, peak at 48 h and are higher in patients with the periodontal disease when compared to healthy controls. For both groups, periostin levels returned to baseline levels within 2 weeks. This transient local increase in periostin in the GCF after eliminating the chronic inflammatory stimuli and bacterial challenge by periodontal surgery in periodontally affected sites suggests the potential role of periostin in the maturation and stability of the connective tissue [78]. Similar results were also reported in healthy patients and patients with chronic periodontitis post nonsurgical low-level laser therapy [79].

#### 2.1.5. Oral Pathology

Periostin has also been identified in several pathological, non-neoplastic conditions, including our analysis of the stroma and differentiated odontogenic epithelium of human ameloblastic fibromas, ameloblastic fibro-odontomas and odontomas [80], in peripheral and central ossifying fibromas, focal cemento-osseus dysplasia, fibrous dysplasia [80], oral lichen planus [81], and ameloblastomas [82].

Studies on periostin expression in human cancers have demonstrated an increased expression in a variety of solid tumours where it is associated with their malignant behaviour (Extensively reviewed by: [83,84]). Specifically in oral leukoplakia [85] and head and neck squamous cell carcinomas (HNSCCs), increased periostin expression appears to be directly proportional to the invasiveness and aggression of the disease [86,87,88,89]. The serum periostin level in head and neck squamous cell carcinoma patients correlates well with that of VEGF-C and with malignant tumour behaviour, including increased tumour stage and lymph node metastasis. Periostin-promoted lymphangiogenesis is mediated by the increased secretion of VEGF-C in cancer cells and by migration and tube formation via Src and Akt activity [90]. TGF-β3 signalling promotes periostin expression by cancer-associated fibroblasts (CAFs), resulting in growth, migration and invasion of cancer cells [89]. Periostin secreted by CAFs also promotes cancer stemness in HNSCCs by activating protein tyrosine kinase 7 [91]. Furthermore, bone marrow mesenchymal stem cells promoted proliferation, invasion, survival, tumorigenicity and migration of head and neck cancer through periostin-mediated PI3K/Akt/mTOR activation [92]. Based on these findings, periostin could be considered a biomarker with prognostic value of malignant behaviours in HNSCC and a potential target for future therapeutic intervention of HNSCC patients [93].

### 2.2. βigh3

TGF-β-induced protein (βigh3) and periostin are considered paralogs because of their structural similarity, as they both contain a single emilin (EMI) and four fasciclin-1 (FAS1) domains. Similar to periostin, βigh3 is also induced by TGFβ signalling in areas of tissue injury and is secreted by activated fibroblasts in the ECM [94]. βigh3 binds directly to collagens type I, II and IV [95] as well as to proteoglycans, such as biglycan and decorin [96], where it modulates cell growth, adhesion and migration, tumorigenesis, wound healing, and apoptosis [97]. βigh3 is expressed in various tissues, including the bone, cartilage, cornea, heart, liver, and skin [98,99].

#### 2.2.1. Development

During murine embryogenesis, βigh3 is expressed in craniofacial cartilage and growth plates [100] and the developing tongue [101]. Han and colleagues found that βigh3 plays a role in chondrocyte and osteoblast differentiation during endochondral ossification [102]. Genetic deletion of βigh3 in *Tgfbi* knockout mice resulted in reduced skeletal size and alterations in cartilage and bone, characterized by matrix degradation [103].

#### 2.2.2. Postnatal Tissues

Recently we showed that *bigh3* mRNA signal is present in the normal, uninjured palate and is upregulated during wound healing. βigh3 message is present in the granulation tissue by day 3 post wounding, with mRNA levels peaking at day 6 after which they are gradually reduced [66]. βigh3 is also expressed in human PDL tissues. In vitro mineralization assay of hPLD showed that exogenous addition of βigh3 has an inhibitory effect on their mineralization [104]. The same research group also showed that during experimental tooth movement of human premolars, βigh3 expression was significantly increased in the PDL [105]. Recently, βigh3 was also found to be expressed in rat pulp, where it could be associated with reparative dentinogenesis pointing to the potential therapeutic role of βigh3 in the pulpal repair process [106].

### 2.3. Matrix Gla Protein

Matrix Gla protein (MGP) is a secreted 14 kDa protein that belongs to a family of γ-carboxylated glutamic acid (Gla)-containing proteins and was initially identified in the demineralized bovine bone matrix [107]. It was later found to be widely distributed in bone, chondrocytes in cartilaginous tissue, associated with vascular smooth muscle cells in the cardiovascular system and kidneys, and by the endothelial-like cells of the trabecular meshwork of the eye [108]. There are five Gla residues in human MGP (four in mice) that undergo γ-carboxylation. Although substantial evidence suggests that MGP may participate in the process of bone metabolism and regulation of osteogenesis, the detailed mechanism of action is still unclear [109,110].

#### 2.3.1. Development

In humans, mutations in the *MGP* gene lead to Keutel syndrome, an autosomal recessive hereditary disorder that is characterized by cartilage calcification, multiple peripheral pulmonary stenoses and severe midfacial hypoplasia with class III malocclusion, accompanied by short stature and brachytelephalangia [111]. Other features of this syndrome are hearing loss, cerebral and tracheal calcifications, and aortic aneurysms. Long-term observation of patients with Keutel syndrome also revealed permanent skin rashes, papillary microcarcinoma of the thyroid, asthma, massive bullous pulmonary emphysema, severe systemic arterial hypertension, and short-term memory loss [112]. The *Mgp* knockout mouse recapitulates all the Keutel syndrome features, including the midface hypoplasia [113]. Generating and subsequently using conditional knock-in and knockout mouse models, Murshed and colleagues found that overexpression of *Mgp* in bone results in mild impairment of bone mineralization. On the contrary, the elevation of *MGP* serum levels did not affect the mineralization of ECM, indicating that ECM mineralization is regulated locally [114]. Despite the craniofacial phenotype of the Keutel syndrome patients and the analogous mouse models, the exact mechanism of MGP’s action in the regulation of craniofacial development is still unknown [110,113].

#### 2.3.2. Postnatal Tissues

In the periodontium, MGP mRNA signal was observed in rat acellular cementum, polygonal PDL cells in the interface between acellular cementum and the uncalcified cellular cementum, suggesting the expression of MGP in the cells adjacent to the cementum may be important to prevent hyper-calcification [115]. During tooth development, data about the expression of MGP are sparse, related only to Argyrosomus regius fish. In this fish, MGP was found to be expressed only in the odontoblastic and ameloblastic processes [116]. In adult human tissues, to investigate changes in gene expression profiles in heathy and diseased pulp, mRNA from clinically healthy and severely carious dental pulp tissues was subjected to oligonucleotide microarrays. In the diseased pulp, MGP levels were found to be upregulated, potentially indicating that MGP participates in the defence and/or repair process of pulp tissue in response to microbial insult [117]. Even though these studies provide supporting evidence of the expression levels of MGP in the pulp, further studies are needed to determine its significance and its role in the development, maintenance and repair of human pulp tissues.

#### 2.3.3. Oral Pathology

Contrary to periostin, MGP has not attracted much attention in the field of oral pathology and neoplasia. Even though the aberrant expression of MGP has been associated with disease progression and poor prognosis in a variety of neoplasms [4], to date, there are no reports on the presence, level of expression and potential roles of MGP in pathological conditions in the oral cavity.

## 3. CCN Family

The CCN family of proteins comprises the following six members: CCN1/CYR61, CCN2/CTGF, CCN3/NOV, CCN4/WISP1, CCN5/WISP2, and CCN6/WISP3. The acronym CCN is derived from the initial letters of cysteine-rich protein 61 (CYR61), Connective tissue growth factor (CTGF), and nephroblastoma overexpressed (NOV/CCN3); these three molecules correspond to CCN1, CCN2, and CCN3, respectively, according to the recent nomenclature [118]. Among them, CCN2 is the most extensively investigated for its function. CCN2 is expressed in not only a variety of cells, including fibroblasts, endothelial cells, chondrocytes, osteoblasts, and smooth muscle cells under physiological conditions but also some inflammatory cells and tumour cells under pathological conditions [119,120]. CCN2 exerts multiple functions under both physiological and pathological conditions in the oral cavity.

### 3.1. Development

As mentioned above, tooth development is characterized by a series of well-regulated, successive reciprocal epithelial-mesenchymal interactions [121], and CCN genes are spatiotemporally regulated during this process. During tooth morphogenesis in mice, Ctgf/CCN2 is expressed in the dental lamina, the inner and outer dental epithelium, as well as the primary enamel knot, the pre-ameloblasts and the condensing mesenchyme at the bud stage [122]. Loss-of-function experiments in organ cultures revealed that CCN2 is essential for both dental epithelial and mesenchymal lineage proliferation and differentiation [122,123,124]. More recent studies found that other CCN proteins are also expressed in the developing tooth germs [125].

During head and orofacial structure development, bones of the cranial vault and the facial skeleton are formed by intramembranous ossification, while the bones that form the base of the skull are formed by endochondral ossification. CCN2 has been shown to participate in both intramembranous and endochondral ossification in mice [126]. The mandible, for instance, is formed by intramembranous ossification of the fibrous mesenchymal tissue around the Meckel cartilage, which acts as a cartilaginous scaffold [127]. In vivo observations in mice and in vitro experiments showed that CCN2 is expressed in highly dynamic manners in developing Meckel’s cartilage where it may influence multiple events, including chondrogenic cell differentiation and chondrocyte maturation, as shown by its strong presence in the condensing mesenchyme around incipient Meckel’s cartilage and along the chondro-perichondrial border. These functions are reported to be downstream of TGFβ and involve stimulating intercellular interactions and expression of condensation-associated genes [128]. In support of these observations, genetic deletion of CCN2 in transgenic mice results in significantly reduced levels of bone matrix synthesis- in particular collagen type I- and osteoblast proliferation, maturation and mineralization in vivo, resulting in decreased skull length with increased skull width. Additionally, osteoblasts isolated from CCN-null mice displayed markedly reduced osteogenic gene expression, impaired proliferation and maturation and consequently delayed mineralization, which was rescued by the exogenous addition of CCN2 [129]. CCN2-null mice also exhibit complete palatal clefting at 100% penetrance [14]. Loss of CCN2 negatively affects the growth, elevation and fusion of the palatal shelves in vivo, while the absence of CCN2 in palatal mesenchymal cells results in reduced proliferation and impaired cell adhesion and spreading, related to an inability to activate Rac1 and RhoA. These effects were again rescued by the exogenous addition of CCN2 [130,131]. Together, these data further highlight the biological significance of CCN2 in orofacial bone development. (In-depth review of the roles of CCN proteins in the orofacial tissues by Kubota [126])

### 3.2. Postnatal Tissues

CCN2 is contributing in various degrees to the wound-healing process in the oral cavity: from the oral mucosa and the gingival tissue to the response of odontoblasts during reparative dentinogenesis. During alveolar bone healing in rats, CCN2 is strongly expressed in the endothelial cells migrating into the granulation tissue formed in the tooth extraction socket [132]. In vitro evidence from gingival- and PDL-derived fibroblasts suggests that TGFβ activates CCN2 expression, which results in enhanced collagen type I synthesis via integrins α6 and β1, through JNK and ALP5 [133,134,135]. Other studies have shown that the application of mechanical tension in PDL cells resulted in increased CCN2 expression and revealed a biphasic effect of CCN2 in these cells, which was dependent on the environmental conditions, suggesting the CTGF/CCN2 might contribute to the preservation of the structural integrity of PDL tissue [136].

While under normal conditions, CCN2 is not expressed in gingival fibroblasts, its expression is induced by the potent profibrotic cytokine, TGFβ, and is overexpressed in fibrotic gingiva in DIGH cases, in both the epithelium and connective tissue [137,138,139,140]. Apart from medication-induced gingival fibrosis, nicotine has also been implicated in periodontal fibrosis. In vitro, the addition of nicotine to gingival and PDL fibroblasts resulted in increased CCN2 protein secretion and collagen type I deposition, effects that were neutralized by anti-CCN2 antibody [141].

CCN2 is not only essential during odontogenesis, but it has also been shown to participate in reparative dentinogenesis in the adult mature pulp [142]. While CCN2 signals are not detected in adult healthy pulp, in response to carious insult, CCN2 expression, as well as BMP-1, is increased in odontoblast-like cells beneath the reparative dentin in carious teeth [143]. Both soluble and immobilized CCN2 protein promoted proliferation, odontogenic gene expression and mineralization of odontoblast-like cells [144], suggesting the potential role of CCN in reparative dentinogenesis by promoting tertiary dentin formation [142] via dynamin-related cellular uptake of BMP-1 [145].

### 3.3. Oral Pathology

Similar to other matricellular proteins, the CCN family of proteins has been shown to regulate key aspects of tumour biology, including proliferation, invasion, matrix remodelling, and dissemination to pre-metastatic niches in distant organs in various neoplastic conditions, and has been associated with poor prognosis [4].

In oral squamous cell carcinoma (SCC), in vitro overexpression of CCN1/Cyr61 appears to regulate the invasive phenotype of these cells [146], and increased expression of CCN1 in human biopsies of oral SCC correlated with poor prognosis of the disease [147]. Additionally, in vitro and in vivo xenograft model experiments provided further support for the aggressive nature of the tumours with CCN1 overexpression [147]. In salivary adenoid cystic carcinoma tissues, CCN1 expression is positively correlated with the histopathological features of the tumours and is associated with advanced disease stages, metastasis in distant organs and overall poor prognosis [148].

Unlike in the case of CCN1, overexpression of CTGF/CCN2 is negatively correlated with oral SCC aggressiveness, thus promoting a favourable prognosis of the disease [149]. Interestingly, loss of CCN2 expression in the late stages of the tumour promotes migration through a switch to N-cadherin expression and epithelial-mesenchymal transition [150], suggesting a role in tumour progression [151]. Ameloblastoma, the most common odontogenic epithelial neoplasm of the jaws, is a benign and slow-growing tumour but is locally aggressive [152]. It was recently shown that parenchyma–stromal CCN2 overexpression positively correlated with fibrous-type stroma, suggesting a role of CCN2 in fibrosis induction in ameloblastoma. Interestingly, the myxoid type of stroma within the same samples exhibited reduced CCN2 expression with decreased collagen deposition [153].

Wingless/Integrated-1(WNT1)-inducible signalling pathway protein 1(WISP-1)/CCN4 induces migration in oral SCC cells by ICAM-1 upregulation via αvβ3 integrin, ASK1, JNK/p38, and AP-1 signalling pathways, and its overexpression is strongly associated with advanced tumour stage [154], tumour progression and treatment failure [155]. Later, the same research group found that tumour secreted WISP-1/CCN4 promotes angiogenesis in oral SCC by upregulating VEGF-A via the αvβ3/FAK/c-Src pathway, which transactivates the EGFR/ERK/HIF1-α signalling pathway in oral SCC cells and increases angiogenesis-related tumour growth in vivo [156]. Further, WISP-1/CCN4 promotes VEGF-C dependent lymphangiogenesis by inhibiting miR-300 (3′-UTR binding repressor of VEGF-C) in vitro via the integrin αvβ3/integrin-linked kinase (ILK)/Akt signalling pathway [157], and it has been suggested as a potential biomarker for predicting lymph node metastasis [158]. Limited evidence exists about the role of WISP-2/CCN5 in head and neck cancer. In salivary gland tumours, WISP-2/CCN5 expression is reduced when compared to normal salivary gland tissue, suggesting that loss of CCN5 may be associated with the development or progression of salivary gland tumours [159]. Similar effects of CCN5 are also observed in oral SCC, where overexpression of CCN5 in oral SCC cells significantly reduced viable cell number, arrested cell cycle, and suppressed cell-cycle regulators (cyclin D1, cyclin E, and CDK2) in vitro, and suppresses tumorigenesis of oral SCC cells in vivo in a xenograft animal model. Collectively, these data suggest that CCN5 could act as a tumour suppressor in SCC via inhibition of the PI3K/AKT signalling pathway [160].

## 4. Thrombospondins

The thrombospondin (TSP) family contains five members (TSP1–5) that represent large, multimeric calcium-binding glycoproteins, which interact with other ECM proteins and contribute to cell-matrix interactions. TSPs are divided into two groups; one contains trimeric proteins, thrombospondin-1 (TSP1) and -2 (TSP2), and the other contains pentameric proteins, TSP3, TSP4 and TSP5 (also known as cartilage oligomeric matrix protein, COMP). TSP1 and TSP2 have received most of the research focus, and they have been associated with various functions across different tissues [4,161,162]. In this section, we will review the roles of TSPs in orofacial and dental development, in the maintenance and repair of postnatal tissues of the oral cavity, and in neoplastic conditions.

### 4.1. Development

Using in situ hybridization and RNAse protection assays, the expressions of TSP1-3 genes were extensively studied in murine embryogenesis [163]. In terms of the orofacial tissues, TSP1 and TSP2 are both present in the head mesenchyme in areas that represent centres of intramembranous ossification [137,163]. Specifically at E15, TSP2 is evident in the nasal septum, developing mandible and Meckel’s cartilage [164]. Increased TSP2 signal is also observed in the developing tooth germ, the mesenchyme contributing to the future PDL, as well as the alveolar bone and periosteum [163]. In newborn mice, the TSP1 signal is lost, while a strong TSP2 signal persists in the developing mandibles [137]. TSP2^−/−^ adult mice displayed significant enamel loss due to mastication forces (attrition) when compared to wild-type age-matched mice, as well as diminished crown width in molars and length of the incisors, as a result of disorganization of the enamel rod architecture, suggesting that TSP2 plays an important role in regulating cell-matrix interactions during enamel formation [165].

### 4.2. Postnatal Tissues

Very sparse data is available regarding the expression of TSPs in adult dental tissues. Ueno and colleagues found that TSP-1 was present only at the position of predentin in anterior bovine teeth. Northern blot analysis revealed high levels of two sizes of TSP1 mRNAs in odontoblasts but not within the dental pulp and gingiva [166]. In vitro, rat pulp cells responded to mineralization factors (such as TGFβ) with increased expression of TSP, as well as osteopontin (OPN), suggesting that TSP might participate in reparative dentinogenesis [167]. Similar observations are reported when hDPSCs were exposed to mineralization conditions, which resulted in a three-fold increase in TSP-1 mRNA levels [138].

In periodontal disease, TSP-1 mRNA levels were found to be significantly increased when compared to healthy gingival tissues. TSP-1 production was dependent upon TLR2/NF-κB signalling and was enhanced by T-cell cytokines, indicating that TSP-1 expression may play an important role in the progression of periodontitis [168]. In response to injury after periodontal surgery, crevicular fluid levels of TSP1 increase, peaking at 72 hours post-surgery and returning to baseline levels after 2 weeks [139]. However, more studies are needed to elucidate the potential role of TPSs in gingival wound healing. In DIGH, a fibrotic condition of the gingiva due to medications, reduction in TSP2 expression was observed in rat and human tissues, as well as HGF cultures, treated by cyclosporin A, in a dose- and time-dependent manner. These findings could indicate a potential protective role of TSP2 against gingival overgrowth, as it is reduced expression and loss of its subsequent angiostatic function might contribute to increased gingival vascularization in DIGH [140]. This data is still quite preliminary, and further studies are needed to determine TSPs roles in DIGH, as well as their potential use as therapeutic agents. Lastly, TSP4 has been studied in the context of neuropathic peripheral pain, where in response to peripheral nerve injury, its expression is upregulated at the dorsal spinal cord [169]. In orofacial neuropathic pain, infraorbital nerve injury leads to TSP4 upregulation in the trigeminal spinal complex in a rat model. Blocking this pathway and reversing TSP4 upregulation may provide a novel therapeutic option for managing trigeminal nerve injury-induced orofacial neuropathic pain states [170].

### 4.3. Oral Pathology

TSP1 is known to be involved in the inhibition of angiogenesis and tumorigenesis in various types of cancer. Expression of TSP-1 in various cancers is significantly downregulated in comparison to normal cells, whereas re-expression of TSP-1 in tumour cells has been associated with anti-angiogenic effects and restriction in tumour size [171,172,173]. In normal oral mucosa, TSP-1 is mainly localized in the subepithelial connective tissue, and in tissues from oral squamous cell carcinomas (OSCCs), TSP-1 intense immunoreactivity is observed in the tumour stroma. In vitro, it was suggested that TSP-1 is mainly produced by fibroblasts, not by epithelial cells, and the synthesis of TSP-1 may be regulated by its interaction with SCC cells [174]. In another study, increased expression of TSP-1 in OSCCs was associated with the reduced micro-vessel density of the tumour and inversely correlated with the pattern of tumour invasion and lymph nodal status, suggesting that TSP-1 has an inhibitory effect on tumour vascularity in OSCCs [175]. TSP-1 has been identified as the most abundantly secreted protein by OSCCs and was reported to be significantly upregulated in OSCC compared to normal tissues where it may promote migration of cancer cells and stimulate the expression of MMPs partly through integrin signalling, thereby favouring OSCC invasion [176]. Recently, it was found that TSP-1 derived from OSCC exosomes participated in the activation of macrophages to an M1-like tumour-associated-macrophages phenotype via p38, Akt, and SAPK/JNK signalling [177]. Additionally, using proteomic profiling of OSCCs, several markers have been identified for their potential prognostic value and correlated with clinicopathological characteristics; among them is TSP-2. A lower abundance of TSP-2 in the invasive tumour front, when compared to the inner tumour area, was correlated with lymph node reoccurrence [178].

## 5. Secreted Protein Acidic and Rich in Cysteine/Osteonectin (SPARC) Family

Secreted protein acidic and rich in cysteine (SPARC), also known as osteonectin or basement membrane protein 40 (BM-40), is a 32 kDa calcium-binding matricellular glycoprotein [179]. SPARC expression is closely associated with that of fibrillar collagens such as collagen type I and functions more as a regulator of cell behaviour rather than a structural part of the ECM, involved in tissue remodelling, repair, development, and cell turnover [180].

### 5.1. Development

SPARC reactivity during development is usually present in tissues undergoing rapid proliferation [181]. In developing teeth of humans, SPARC is first detected at the bell stage of the tooth germ intracellularly in odontoblasts and was linked to the production of dentin, whereas ameloblasts, undifferentiated dental pulp mesenchymal cells [182], the inner and outer enamel epithelium, cells of the stellate reticulum and stratum intermedium, enamel, dentin and cementum layers showed no expression of SPARC [183]. SPARC signal was also observed in active intramembranous ossification sites at the areas of newly formed bone but not in the osteocytes of the human embryonic mandibles [181]. An extensive study completed upon human tissues from fetal stages of development to adult tissues, SPARC was found to be expressed in the initial stages of cytodifferentiation in the teeth. At the maturation stage of enamel formation, SPARC was detected in odontoblasts and their processes within the extracellular matrix and was mostly present in the nonmineralized predentin [184].

### 5.2. Postnatal Tissues

SPARC is expressed at high levels in osseous tissues with high turnovers, such as in active osteoblasts and bone marrow progenitor cells, odontoblasts, PDL fibroblasts, hypertrophic chondrocytes, and osteoid [185,186,187]. The function of SPARC in mineralized tissues both during homeostasis and disease has yet to be fully defined; however, SPARC-null mice showed the importance of SPARC for normal remodelling and maintenance of bone mass, as these mice showed to have osteopenia, decreased osteoblast number and bone formation rate, due to reduced osteoblast formation, maturation, and survival [182]. (For an in-depth review of the role of SPARC in mineralized tissues, refer to [188]). Recent evidence suggests that SPARC regulates the ECM mineralization of osteoblasts through the P38 signalling pathway [189]. SPARC-null mice also exhibit reduced amounts of fibrillar collagen in the PDL, where the PDL fibres demonstrate differences in morphology characterized by lower amounts of thick collagen fibres versus WT PDL [190]. When the PDL of SPARC-null mice was challenged with bacterial LPS, they exhibited greater loss of PDL collagen fibres in comparison to WT in the absence of increased inflammatory cell infiltration [191]. Thus, the reduction in size and number of collagen fibres in SPARC-null PDL appears to result in increased susceptibility to periodontal disease, despite a reduction in inflammatory mediators [191] and decreased mechanical strength [192]. These effects were rescued by inhibition of transglutaminase activity (extracellular crosslinking protein), providing evidence for a function of transglutaminase in fibrillar collagen assembly in vivo and the regulation of this activity by SPARC in rat PDL [192].

Recently, using whole-exome sequencing, mutations in the gene encoding SPARC were identified in two individuals with recessive osteogenesis imperfecta (OI) type IV [193]. Osteogenesis imperfecta, or “brittle bone disease”, is a hereditary connective tissue disorder, most often is caused by mutations in COL1A1 or COL1A2 genes, leading to abnormalities in collagen primary structure, insufficient quantity, abnormal post-translational modification, folding, intracellular transport or matrix incorporation [194,195]. The clinical presentation of OI includes low bone mass and reduced bone material strength, resulting in bone fragility and easy susceptibility to fracture, bone deformity and growth deficiency, tooth abnormalities (dentinogenesis imperfecta) and often blue or grey sclera. In this recent report, mutations in the gene encoding SPARC in individuals with OI were found to be on the sequences of the collagen-binding site, resulting in a reduced SPARC affinity for collagen I [193].

In the human dental pulp, SPARC is expressed by the post-mitotic, terminally differentiated odontoblasts, as well as by in vitro-differentiated odontoblast-like cells [161], particularly after they reach confluence [162]. Looking into the effect of various growth factors and cytokines on the transcription and protein levels of SPARC in dental pulp cells in culture, Shiba and colleagues found that the addition of TGFβ induced SPARC expression levels, while basic FGF (bFGF), platelet-derived growth factor (PDGF), epidermal growth factor (EGF), and TNF-α and IL-1β cytokines decreased SPARC and alkaline phosphatase (ALP) levels [162]. Based on the observation that TGFβ induced SPARC synthesis before evidence of calcification in vitro, they suggested that SPARC may play some roles before the calcification of the pulp matrix in the remodelling of the dental matrix, as well as calcification during odontogenic differentiation [162].

### 5.3. Oral Pathology

SPARC has been shown to play significant roles in tumorigenesis, altering cancer cell activity and the tumour’s microenvironment, modulating cell growth, apoptosis, adhesion migration and invasion, regulating ECM and the activity of matrix metalloproteinases [196,197]. However, the role of SPARC in cancer research has been the subject of controversy and appears to be context and tissue-dependent [197]. In the oral cavity, increased SPARC immunoreactivity on the tumour stroma and the tumour cells in OSCCs has been positively correlated with the presence of distant metastases and patient survival, indicating that SPARC could have a prognostic role of in OSCC [198]. In a more recent study, even though significantly more SPARC-positive cells were present in oral leukoplakia, carcinoma in situ, and early invasive OSCC biopsies when compared to normal mucosal epithelium, there were no correlations between SPARC immunoreactivity and prognosis of invasive oral SCCs [199]. SPARC has also been found to be expressed in the margin between the primary tumour and the surrounding microenvironment (stroma cells) in primary and regional metastatic oral tongue squamous cell carcinomas, suggesting its role in invasion and metastasis [200]. Recent studies report SPARC expression by neoplastic cells in epithelioid osteosarcoma of the mandible [201] and the ECM of mucoepidermoid carcinoma of salivary glands where its expression is associated with low histological grade tumour [202].

## 6. SIBLING Family

Small integrin-binding ligand N-linked glycoproteins (SIBLINGs) are a family of glycophosphoproteins that includes bone sialoprotein (BSP), dentin sialophosphoprotein (DSPP), dentin matrix protein-1 (DMP1), osteopontin (OPN), and matrix extracellular phosphoglycoprotein. Structurally, they all contain an Arg-Gly-Asp (RGD) integrin-binding motif that facilitates cell attachment, migration, differentiation and triggers intracellular signal transduction via binding to cell surface receptors, such as integrin, and functionally they overall activate specific MMPs to mediate processes involved in ECM degradation and are best described in mature, mineralized tissues [203]. Even though common this family of proteins share similar characteristics, they have been reported to display differential tissue distributions and functions that are highly dependent on their post-translational modifications [204]. In this review, we will briefly summarize what is known to date about the roles of SIBLING proteins in the development and pathology of dental tissues.

### 6.1. Bone Sialoprotein

Bone sialoprotein (BSP) is an anionic phosphoprotein primarily found in mineralized tissues, such as bone, calcified cartilage, dentin, and cementum. It is known to have essential roles in the initial formation of hydroxyapatite at the onset of mineralization [205]. BSP binds collagen and hydroxyapatite with high affinity [206] and promotes mineral formation [207]. Loss of BSP in transgenic mice revealed the essential roles in bone growth and mineralization [205], characterized by reductions in bone mineral density, bone turnover, osteoclast activation, impaired bone healing, and disorganization of the periodontal ligament. The lack of acellular cementum in BSP-null resulted in progressive loss of periodontal attachment, extensive alveolar bone and tooth root resorption, and incisor malocclusion [208,209]. When the masticatory and occlusal forces were reduced by placing the BSP-null mice on a soft diet, some of the phenotypical characteristics, such as body weight, long bone length, and serum alkaline phosphatase activity were rescued. This suggested that tooth dysfunction and malnutrition contribute to the growth and skeletal defects reported in these mice [210]. A recent study showed that in BSP-null mice, osteopontin is overexpressed, complicating the understanding of their phenotype [211]. While the significance of BSP in the processes of cementogenesis and intramembranous ossification of craniofacial bones is well established, loss of BSP in BSP-null mice has a minimal effect on endochondral ossification in the cranial base and does not affect dentinogenesis in BSP-null teeth. Dissimilar effects of loss of BSP on mineralization of dental and craniofacial tissues suggest site- and tissue-specific effects of BSP or yet to be defined interactions with site-specific factors [208].

### 6.2. Osteopontin

Osteopontin (OPN) is 44 kDa acidic arginine-glycine-aspartate containing adhesive glycoprotein, which is mainly synthesized by osteoblasts, osteocytes and other hematopoietic cells. In addition, OPN is secreted by immune cells such as neutrophils, dendritic cells, NK cells, T cells and B cells [212]. Extensive evidence shows that OPN plays a role in bone remodelling, anchoring osteoclasts to the mineral matrix of bones, and the regulation of both the innate and adaptive immune systems, promoting a cell-mediated immune response [213].

In murine neonatal tissues, OPN signal is present in numerous osteoblasts of the alveolar bone, new cementoblasts near the apical root, and some odontoblasts. At a later timepoint, 1 month postnatally, OPN is expressed in bone cells, few cementoblasts along the acellular cementum, many cementoblasts surrounding the cellular cementum, and cementocytes in the cellular cementum [214]. This study also showed that OPN regulates the formation and mineralization of dentin and bone, influences tissue properties of PDL and pulp, but does not control acellular cementum apposition [214]. The functional role of OPN has been studied in transgenic mice. OPN-null mice display impaired resorption, and OPN is described as an inhibitor of mineralization but without presenting phenotypical abnormalities in skeletal development and osteogenesis [215]. A recent micro-CT analysis of dental tissues in OPN-null mice revealed significantly increased volumes of dentin/cementum, mandibular bone, and enamel when compared to wild-type mice. As a consequence of increased dentin/cementum volume, the dental pulp space volume, as well as the PDL space, in these mice are significantly decreased. Regarding the role of OPN in the dentin-pulp complex, OPN is detected in the dentin and the boundary between the reparative and secondary dentin in rats [216]. A recent study revealed that the deposition of OPN at the calcification front during the pulpal repair process is essential for the secretion of type I collagen by newly differentiated odontoblast-like cells, needed to form reparative dentin [185]. They also showed that odontoblast-like cell differentiation, after the death of primary, post-mitotic odontoblasts due to injury, was not affected in OPN-null mice. This suggested that OPN might not be essential for the differentiation of odontoblast-like cells, although dentin matrix protein 1 likely compensates for OPN in this process [217].

OPN is expressed in numerous carcinomas and plays a role in tumour development, invasion, and metastasis. In the oral cavity, OPN is not normally detected in healthy epithelium/mucosa [218]. However, it is upregulated in premalignant and malignant lesions arising from oral epithelium, such as OSCCs, with the highest levels detected in the plasma or the tumour and is associated with tumour progression, suggesting that OPN expression is an important prognostic factor for OSCC [219], resistance to treatment, and poor survival [220]. In a recent study, reduction in OPN in OSCC lines in vitro resulted in suppression of cell proliferation, colony formation and in vivo tumorigenic ability of OSCC cells. They also showed that OPN is a downstream target of mTOR complex 1, suggesting that mTORC1 and OPN might be potential therapeutic targets for OSCCs with aberrant mTORC1 signalling [221]. OPN is also upregulated in salivary gland tumours, such as pleomorphic adenoma, adenoid cystic carcinoma, and polymorphous low-grade adenocarcinoma [222,223], with higher expression rates observed in acinic cell adenocarcinoma and mucoepidermoid carcinomas [186]. While the role of OPN in tumorigenesis in head and neck cancer is not fully understood, evidence indicates that this protein may induce the malignant phenotype of cells by activation of the PI3K/AKT/mTOR pathway, which favours cell proliferation, invasion, metastasis, angiogenesis, and failure of treatment [187].

### 6.3. Dentin Sialophosphoprotein

Dentin sialophosphoprotein (DSPP) is a dentin extracellular matrix protein that is processed into dentin sialoprotein (DSP), dentin glycoprotein (DGP) and dentin phosphoprotein (DPP) [224]. DSPP/DSP is mainly expressed in odontoblasts and preameloblasts [225], but mRNA transcripts and protein signals are also found in rat alveolar bone, cellular cementum, osteocytes, cementocytes, and their matrices [226]. Evidence suggests that DSP and peptides derived from DSP regulate gene expression and protein phosphorylation, induce dental primary/stem cell differentiation, and play a crucial role in dentin and bone mineralization [227]. Additionally, it has been shown that DPP in the dentin matrix activates AKT and the mTOR signalling pathway to promote preodontoblast survival and differentiation [228]. DSP- null mice demonstrate phenotypes similar to the manifestations of human dentinogenesis imperfecta type III, exhibiting defects in dentin and bone mineralization [229]. Hereditary dentin disorders, dentinogenesis imperfecta type II/III and dentine dysplasia, are currently proposed to be one disease with distinct clinical manifestations reflecting various mutations in the same DSPP gene [230]. This indicates that DSPP is critical for dentin mineralization. Although the exact mechanisms by which DSPP participates in biomineralization are unclear, it has been hypothesized that the proteolytic processing of DSPP into DPP and DSP is an activation step that converts a large precursor into active fragments directly involved in biomineralization, i.e., the roles of DSPP in biomineralization reside in its cleaved products, DPP and DSP. Analysis of transgenic mice revealed distinct roles of DSP and DPP in dentin mineralization, with DSP regulating the initiation of dentin mineralization and DPP being involved in the maturation of mineralized dentin [229]. DPP forms insoluble aggregates in the presence of Mg++ and Ca++, and this special affinity for calcium is thought to be related to the biological role of DPP in the nucleation and growth of hydroxyapatite crystals during dentin mineralization [231]. Another interesting study suggested that the overexpression of DSPP promoted the mineralization of adipose-derived stem cells (ADSc) along with the expression of early odontogenic marker genes, implying that these cells may differentiate into functional odontoblast-like cells [232].

### 6.4. Dentin Matrix Protein 1

Dentin matrix protein 1 (DMP1) is an acidic, highly phosphorylated non-collagenous protein with an overall amino acid composition between bone phosphoprotein and dentin phosphophoryn, reported to play a regulatory role in the hydroxyapatite crystal growth [233]. Initially, DMP1 was postulated to be dentin specific, but its expression is also detected in osteocytes and osteoblasts involved in the mineralization of dentin and bone [234].

Apart from its role in matrix mineralization, DMP1 is also known to act as both an intra- and extracellular signalling molecule regulating odontoblast differentiation at several stages of tooth development. In human fetal tooth germs, BMP1 expression is present in the dental lamina, as well as in the cells of the external epithelium, stellate reticulum and stratum intermedium of the enamel organ. In the developing crown, DMP1 is expressed in the ameloblast and odontoblast layer, as well as in the dentinal tubules of coronal dentin near the odontoblast area. In erupted, fully formed human teeth immunolabelling for DMP1 is only observed in the dentinal tubules, while the mineralized enamel, predentin and dental pulp matrix are negative [235]. Dmp1 transcripts appeared at the late bud stage of molar morphogenesis, and their levels are decreased in secretory odontoblasts after the appearance of mineral apposition [236] DMP1-null mice exhibit partial failure of maturation of predentin into dentin, enlarged pulp chambers, increased width of predentin zone with reduced dentin wall, and hypomineralization defects [237]. In humans, DMP1 mutations lead to autosomal recessive hypophosphatemic rickets [238]. Chen and colleagues showed that all of the five SIBLING members were expressed within the cytoplasm and cellular processes in the mouse odontoblastic cell lines. Expression levels of DMP1 and DSPP were higher in differentiated mouse odontoblasts than undifferentiated mouse odontoblasts. Immunolabelling signal of DSP and MEPE was also detected within the nucleus in the two cell lines. Western blot assay indicated that all five members were processed into at least two fragments in these cells, suggesting that different processed products and expression levels of the SIBLING proteins may play distinct biological functions in tooth development and mineralization [239].

Regarding the role of DMP1 in reparative dentinogenesis during pulp healing, DMP1 is found to be deposited along the boundary between the primary dentin and the newly formed dentin, and its expression preceded the appearance of nestin-immunoreactive cells (odontoblastic marker), active cell proliferation and new matrix formation, suggesting that DMP1 acts as a trigger of pulp repair [240].

## 7. Tenascin-C

Tenascin-C (TNC) is a large hexameric glycoprotein sharing several features with the extensively investigated molecules such as fibronectin (FN) and laminin (LN), and it is a member of a protein family comprising at least four different molecules in humans: tenascin-C, tenascin-R, tenascin-W, and tenascin-X. Under physiological conditions, TNC is found abundantly in several embryonic tissues, especially related to the epithelial-mesenchymal transition (EMT) and to cell migration pathways. In adult tissues, it is expressed mainly during inflammation, wound healing, and tumorigenesis [241,242].

### 7.1. Development

Early reports suggest that the dental epithelium induces TNC expression in early dental mesenchyme [243]. During tooth development, TNC is transiently expressed during epithelial budding in the condensed dental mesenchyme (dental papilla), particularly at areas of epithelial-mesenchymal interface, but is temporally reduced at the cap stage. In mouse and rat tooth germs, during the bell stage of tooth morphogenesis, TNC signal is intensified and accumulated in the dental pulp even after completion of crown development and eruption but is absent in the dental follicle as well as the dental epithelial cells, including the stellate reticulum. Although TNC signal is present in the dental basement membrane at the time of odontoblast differentiation, TNC is absent in mature, terminally differentiated odontoblasts and the dentin layer [244].

### 7.2. Postnatal Tissues

In postnatal normal oral mucosa, TNC expression is sparsely distributed in a discontinuous manner at the basement membrane zone [241]. Studies in murine lip mucosa in embryonic and adult tissues revealed that in unwounded fetal lip tissues, TNC is present in and below the basement membranes of the oral mucosa and the dermal-epidermal junction, with this pattern being maintained in normal murine neonatal and adult tissues. Upon wounding, TNC expression appears at earlier timepoints in fetal tissues (at 1 h post wounding) when compared to adults (24 h). Tenascin inhibits the cell adhesion effect of fibronectin, and during development, the appearance of TNC correlates with the initiation of cell migration. The authors suggested that the fact that the appearance of TNC preceded cell migration during the wound-healing process along with the rapid closure that fetal wounds display may be due to the early appearance of tenascin in the wound [245]. During wound healing in rat tongue tissues, TNC expression is transiently increased, especially close to the basement membrane zones at the wound edges beneath the proliferating and migrating epithelium. TNC signal intensity and expression pattern are similar during the proliferative phase in the regenerating connective tissue, after which it slowly subsides [246]. A similar expression pattern is also observed in human oral mucosa tissues [247]. TNC transient expression pattern and spatial and temporal coordination with other adhesive molecules (integrins) suggest that these interactions may be among mechanisms that regulate epithelial cell proliferation, migration, and phenotype during wound repair in oral mucosa [247]. Transient expression of TNC is also observed in the injured human dental pulp and the dentinal bridge forming after pulp mechanical injury, indicating a potential role of TNC in the reparative dentinogenesis process [248].

### 7.3. Oral Pathology

In oral lesions, it has been suggested that TNC increased expression is frequently seen in association with inflammation, whether the lesions are hyperkeratotic, dysplastic or neoplastic [249]. TNC has a variety of pro-tumorigenic interactions with cancer cells, cancer-associated fibroblasts, lymphocytes and tumour-associated macrophages, as well as angiogenesis promoting interactions with endothelial cells. It interacts with cancer cells by reducing adhesion, enhancing proliferation, migration and invasion, and increasing dissemination and homing [250,251].

As mentioned above, in normal buccal and palatal mucosa, in the ventral tongue, in the floor of the mouth and in gingiva, TNC immunoreactivity is observed as a continuous thin, delicate line restricted at the basement membrane region. In hyperkeratosis, without dysplasia, a distinct zone of increased TNC immunoreactivity observed immediately beneath the epithelium, and a more intense signal is observed in dysplasias of various degrees, in situ carcinomas and OSCCs [249]. TNC immunoreactivity is more intense at the advancing edges of the tumour, and the triggering factor for TNC expression from the tumour surrounding environment/stroma seems to be of epithelial origin, suggesting TNC’s role in organizing and remodelling the stroma to support active epithelial proliferation and migration [249]. TNC is also upregulated in neoplastic lesions of salivary glands and mostly observed in a less differentiated and higher degree of malignancy tumours, such as solid adenoid cystic carcinomas [252], in oral leukoplakia, lichen planus [253], in oral submucous fibrosis [254] and inflammatory gingival hyperplasia [241]. In a recent study, TNC expression in tumour cells of OSCC of the tongue was not a prognosticator of disease progression. However, stromal TNC and fibronectin expression were found to be independent prognosticators among all stages, and more importantly, among early stages, thus differentiating patients into low- and high-risk groups. The authors suggested that surgery alone of early-stage primary tumours might be adequate when stromal FN is negative, but aggressive treatments should be considered when both TNC and FN are abundant [255].

## 8. Harnessing MCPs for Orofacial and Dental Tissues Regeneration

As discussed in this review, MCPs play significant roles in orofacial and dental healing processes, and their diverse functions make them an important consideration in the design and delivery of materials. In the last decade, many different approaches and biologic agents have been proposed in the area of orofacial engineering [256,257]. Many types of scaffolds have been studied for their potential use in regenerative periodontics and endodontics, such as collagen, hyaluronic acid, polylactic acid, platelet-rich plasma, fibrin, chitosan-based hydrogels and more, with or without various growth factors. The complexity of cell-ECM interactions suggests the need for tissue-specific strategies for restoring native cellular niches [258]. Given that the structural and compositional properties of ECM are unique to each tissue [259], increasing lines of evidence demonstrate that the differences in ECM properties can modulate stem/progenitor cell lineage specification and support the rationale for further investigation of tissue-specific ECM as a cell-instructive component to guide stem/progenitor cell differentiation in regenerative procedures [260,261]. Another major challenge remains the temporal organization of the sequence of events that must take place within the healing area in order to promote the cells that are needed at each precise moment and without compromising normal cell function. With that in mind, novel tailored biomaterials and signalling molecules—whether delivered by gene therapy or not [262,263]—would be of great interest. Lastly, host tissue response to the implanted biomaterials is key to promoting optimal tissue regeneration; thus, the design of biomaterials to specifically alter the levels of MCPs surrounding implants provides a new avenue for the design and fabrication of biomimetic biomaterials [264].

## 9. Conclusions

In this review, we discussed the roles and functions of matricellular proteins in the development and maintenance of dental and orofacial tissues (Table 1). Summarizing the evidence from in vitro studies, in vivo and clinical studies, we also discussed their role in wound-healing processes in the oral mucosa and teeth, as well as oral pathology and tumorigenesis in the neck and head region. Of particular interest for clinical applications is the prognostic value of some MCPs in the progression and outcome of HNSCC. In this review, we have also highlighted the gaps in the literature regarding the unaddressed mechanistic details of some MCPs in dental tissues. The future outlook of MCPs in orofacial and dental tissue regeneration is promising with growing applications in preclinical settings, as briefly highlighted in this review. Identification of the functional domains and pathways responsible for the effects of matricellular proteins will allow the design of biomimetic materials that selectively reproduce specific actions toward tissue regeneration or inhibit detrimental for the wound-healing effects in a controlled temporal manner.

## Figures and Tables

**Table 1:** Summary of matricellular protein expression, transgenic animals’ phenotype, and involvement in disease and pathology in orofacial tissues.

Protein	Expression Sites	Transgenic Mouse Phenotype of Orofacial Tissues		Clinical Presentation of Gene Mutation	Expression Changes in Diseased States	References
		Knockout	Overexpression			
**Periostin**	Dental papilla cells, trans-differentiating odontoblasts, fibroblasts, collagen-rich, biomechanically active tissues, EMT sites during development,	Defective collagen fibrillogenesis Severe periodontal disease Reduction in bone density Structural enamel defects Defective/absent orbital bones	Unknown	Unknown	↑gingival wound healing ↑palatal wound healing ↑in PDL - orthodontic movement force ↑ ameloblastic fibromas, fibro-odontomas odontomas, peripheral and central ossifying fibromas, focal cemento-osseus dysplasia, fibrous dysplasia ↑ lichen planus ↑ positive correlation with invasive OSCCs aggressiveness/poor prognosis	[18,30,32]
**β** **igh3**	Fibroblasts, Osteoblasts, chondrocytes, craniofacial cartilage and growth plates, endochondral ossification sites	Reduction in skeletal size Degradation of the bone matrix	Retinal degeneration	potential risk gene for human diabetes	↓ in PDL - orthodontic movement force	[101,103,105,265,266]
**MGP**	Bone, chondrocytes, vascular smooth muscle cells	Midface hypoplasia Calcification of the spheno-occipital synchondrosis	Hypomineralization of dentoalveolar tissues	Keutel syndrome	Unknown	[110,111,113]
**CCN**	Fibroblasts, endothelial cells, chondrocytes, osteoblasts, and smooth muscles, developing tooth germs, Meckel’s cartilage	Reduced bone matrix synthesis Reduced osteoblast proliferation Decreased skull length, increased skull width Palatal clefting	Decreased bone mineralization and trabecular bone volume Decreased rates of bone formation and mineral apposition	CCN6: autosomal recessive progressive pseudorheumatoid dysplasia	↑ in PDL wound healing ↑ in bone healing in extraction socket ↑ in PDL - orthodontic movement force ↑in reparative dentinogenesis ↑CCN1: positive correlation with invasive OSCCs aggressiveness/poor prognosis ↑CCN2: negative correlation with oral SCC aggressiveness ↑CCN4: positive correlation with lymph node metastasis of OSCCs ↓CCN5 in salivary gland tumours	[126,142,147,149,155,159,267,268]
**TSPs**	Centers of intramembenous ossification, developing nasal septum, mandible, Meckel’s cartilage, tooth germ, bone, periosteum	Defects in collagen fibrillogenesis TSP2^-/-^: enamel defects	Unknown	Thrombotic thrombocytopenic purpura Peters-Plus Syndrome TSP1,TSP4: familial premature myocardial infarction TSP5: autosomal dominant bone dysplasia-pseudoachondroplastic dysplasia	↑ TSP1 in periodontal and healing ↓TSP2 in DIGH tissues ↑TSP4 in neuropathic peripheral pain	[139,163,165,168,269]
**SPARC/ON**	Tooth germs, dentin, developing alveolar bone, intramembranous ossification sites, PDL fibroblasts, osteoblasts, odontoblasts, hypertrophic chondrocytes, and osteoid	Osteopenia Decreased osteoblast number and bone formation rate Reduced amounts of fibrillar collagen in the PDL Susceptibility to periodontal disease	Unknown	Osteogenesis imperfecta type IV	↑ in periodontal disease	[184,188,190,193]
**BSP**	Osteoblasts, osteoclasts, osteocytes, chondrocytes, dentin	Reduced bone mineral density, bone turnover, osteoclast activation Impaired bone healing Disorganization of PDL Lack of acellular cementum Progressive loss of periodontal attachment Extensive alveolar bone and tooth root resorption Incisor malocclusion	Multi-dwarfism Decreased bone mass density Decreased trabecular bone volume	Fibrous Dysplasia Chondromalacia	Controversial- *see main text for details*	[205,208,209,211,270,271,272]
**OPN**	Osteoblasts, osteoclasts, osteocytes, hypertrophic chondrocytes, immune cells, odontoblasts, cementoblasts, cementocytes	Impaired mineral resorption Increased volumes of dentin/cementum, mandibular bone, and enamel	Sjögren’s syndrome phenotype	Unknown	Systemic lupus erythematosus, rheumatoid arthritis, multiple sclerosis, Sjögren’s syndrome, type I diabetes ↑ in OSCCs, salivary gland tumors	[187,214,215,223,227,273]
**DSPP**	Odontoblasts, preameloblasts, osteocytes, cementocytes, dentin, bone, cementum	Defect in dentin mineralisation. Bones display accelerated mineralisation and changes in structural properties	DSP-accelerated mineralisation in teeth DPP-deleterious effects onenamel	Dentinogenesis imperfecta type II/III and dentine dysplasia	↑ in dental pulp wound healing process	[226,230,274]
**DMP1**	Osteoblasts, osteoclasts, osteocytes, hypertrophic chondrocytes, bone, dentin	Partial failure of maturation of predentin into dentin Enlarged pulp chambers Increased width of predentin zone, reduced dentin wall Hypomineralization defects Lower mineral content Defective cartilage formation resembling dwarfism with chondrodysplasia. Hypophosphataemia	Narrow growth plate with accelerated mineralisation and increased bone turnover	Autosomal recessive hypophosphataemic rickets	↑ in dental pulp wound healing process	[235,240,275,276]
**TNC**	Developing tooth germ (dental papilla), dental pulp, fibroblasts, PDL	Not evident pathologic phenotype- unknown in orofacial tissues	Unknown	Unknown	↑ in oral wound healing ↑ in OSCCs, salivary gland tumours	[244,246,247,277]
